# Molecular diversity of *Mycobacterium tuberculosis *isolates from patients with pulmonary tuberculosis in Mozambique

**DOI:** 10.1186/1471-2180-10-195

**Published:** 2010-07-21

**Authors:** Sofia O Viegas, Adelina Machado, Ramona Groenheit, Solomon Ghebremichael, Alexandra Pennhag, Paula S Gudo, Zaina Cuna, Paolo Miotto, Véronique Hill, Tatiana Marrufo, Daniela M Cirillo, Nalin Rastogi, Gunilla Källenius, Tuija Koivula

**Affiliations:** 1 Faculty of Veterinary, Eduardo Mondlane University, Maputo Mozambique; 2 National Institute of Health, Ministry of Health, Maputo, Mozambique; 3 Department of Microbiology, Tumor and Cell Biology, Karolinska Institutet, Stockholm, Sweden; 4 Department of Bacteriology, Swedish Institute for Infectious Disease Control, Solna, Sweden; 5 Tuberculosis National Control Program, Ministry of Health, Maputo, Mozambique; 6 Emerging Bacterial Pathogens Unit, San Raffaele Scientific Institute, Milan, Italy; 7 WHO Supranational TB Reference Laboratory, Tuberculosis & Mycobacteria Unit, Institut Pasteur de la Guadeloupe, Abymes, Guadeloupe, France; 8 Department of Clinical Science and Education, Södersjukhuset, Karolinska Institutet, Stockholm, Sweden; 9 Centre for Microbiological Preparedness, Swedish Institute for Infectious Disease Control, Solna, Sweden

## Abstract

**Background:**

Mozambique is one of the countries with the highest burden of tuberculosis (TB) in Sub-Saharan Africa, and information on the predominant genotypes of *Mycobacterium tuberculosis *circulating in the country are important to better understand the epidemic. This study determined the predominant strain lineages that cause TB in Mozambique.

**Results:**

A total of 445 *M. tuberculosis *isolates from seven different provinces of Mozambique were characterized by spoligotyping and resulting profiles were compared with the international spoligotyping database SITVIT2.

The four most predominant lineages observed were: the Latin-American Mediterranean (LAM, n = 165 or 37%); the East African-Indian (EAI, n = 132 or 29.7%); an evolutionary recent but yet ill-defined T clade, (n = 52 or 11.6%); and the globally-emerging Beijing clone, (n = 31 or 7%). A high spoligotype diversity was found for the EAI, LAM and T lineages.

**Conclusions:**

The TB epidemic in Mozambique is caused by a wide diversity of spoligotypes with predominance of LAM, EAI, T and Beijing lineages.

## Background

Tuberculosis (TB) is one of the major health problems in Mozambique. It is estimated that 27,000 deaths caused by TB occur each year with an estimated incidence and prevalence rate of 431 and 504 per 100,000 population, respectively[1]. Mozambique ranks 19^th ^on the list of 22 TB high-burden countries in the world. A steady increase in the prevalence rate of Human Immunodeficiency Virus (HIV)/Acquired Immune Deficiency Syndrome (AIDS) (up to an estimated 16.2% among the population aged 15 to 49 years in 2004) makes the situation even more precarious. Mozambique, with around 20 million inhabitants, shares geographical borders with six other countries where TB is also endemic, i.e., South Africa, Swaziland, Zimbabwe, Zambia, Malawi and Tanzania.

At present Mozambique has 252 district laboratories performing smear microscopy for TB diagnosis and one National Reference Laboratory that performs culture and drug susceptibility testing of *Mycobacterium tuberculosis *complex (MTC) isolates.

Molecular genotyping is an important tool for the understanding of TB epidemiology. Despite the high TB burden in the Sub-Saharan Africa region, there is currently a paucity of information regarding the genetic diversity of MTC strains in Mozambique and no published data is available.

Various methods have been used for phylogenetic and population genetic studies [2]. Spoligotyping is a Polymerase Chain Reaction (PCR)-based genotyping method that permits the assessment of the MTC genetic biodiversity in a rapid, reliable and cost effective way [3]. On the basis of the variability of the direct-repeat locus [3], spoligotyping has been used worldwide to type large numbers of strains in population based studies.

In the present study, we characterized by spoligotyping 445 MTC isolates from a Drug Resistance Surveillance study performed in Mozambique over a 1-year period (2007-2008), in order to identify the predominant spoligotypes responsible for the prevalence of TB in Mozambique.

## Results

### Patients

**Figure 1:**
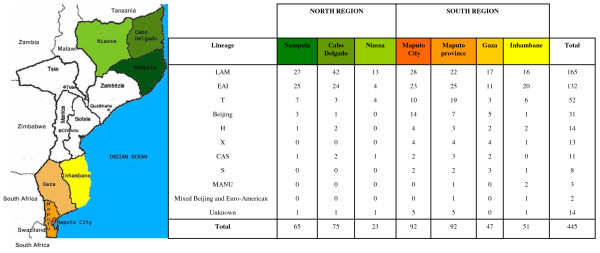
**Geographical distribution of *M. tuberculosis *predominant spoligotype lineages in 7 provinces of Mozambique**. The map describes the geographical distribution of predominant spoligotype lineages in Maputo city, Maputo province, Gaza, Inhambane, Nampula, Cabo Delgado and Niassa. The number of isolates per lineage in each province is depicted. Lineages: Latin American Mediterranean (LAM); East African Indian (EAI); T lineage; Beijing; Haarlem (H) strains; X clade; Central Asian strains (CAS); S lineage, and the "Manu" lineage.

The study included a total of 445 *M. tuberculosis *strains isolated from patients in Mozambique recruited during a resistance surveillance study over a 1-year period (2007-2008). The preliminary results of the Drug Resistance Surveillance study provided by the National Tuberculosis Control Program indicate that 7.8% of all new cases analysed in their sample presented any resistance and 3.5% were multi-drug resistant [4]. Of the isolates included in the present study, 282 were from the South region of the country and 163 were from the North (Fig 1).

The demographic information of the patients showed that 278 (62.5%) were male while 167 (37.5%) were female. The patients' median age was 32 years (SD 13.3) with a range of 15-82 years. Stratification according to age showed that 244 (54.8) of the patients were aged 15-34, 144 (32.4%) were 35-54 years while 44 (9.9%) were 55+. In 13 (2.9%) cases, information about age was not available.

Of all the patients, 98 (22%) were HIV positive, 122 (27.4%) HIV negative and 225 (50.6%) were not tested for HIV. The majority of the HIV positive patients were from the South 89/195 (45.6%), while 9/25 (36.0%) were from the North. The age distribution among patients that were tested for HIV and the ones that were not tested were similar, patient's median age were 32 (SD 13.9) and 31.5 years (SD 12.7) respectively.

### Spoligotyping

Spoligotyping produced a total of 147 different patterns for the 445 strains studied. Forty-nine patterns corresponded to orphan strains that were unique among more than 73,000 strains recorded in the SITVIT2 database (Additional file 1), as opposed to 98 patterns from 396 patients that corresponded to shared-types (SITs), i.e. an identical pattern shared by two or more patients worldwide (within this study, or matching another strain in the SITVIT2 database), as shown in Additional file 2. The genotypic clade designations, the percentage distribution of all SITs observed in this study; for each of the SIT shown, their binary/octal description, the number of total strains and percentage in the present study as compared to the same in the SITVIT2 database are summarized in Additional file 2. Phylogenetic lineage description for each SIT was also provided. For the 98 SITs recorded a total of 79 SITs (containing 368 isolates) matched a pre-existing SIT in the SITVIT2 database, whereas 19 SITs (containing 28 isolates) were newly-created either within the present study or after a match with an orphan in the database. Irrespective of the database comparison, 50 patterns corresponded to clusters in the present study (Additional file 2); 50 clusters containing 348 isolates (2 - 32 isolates per cluster), amounting to an overall clustering rate of 78.2% (348/445).

**Figure 2:**
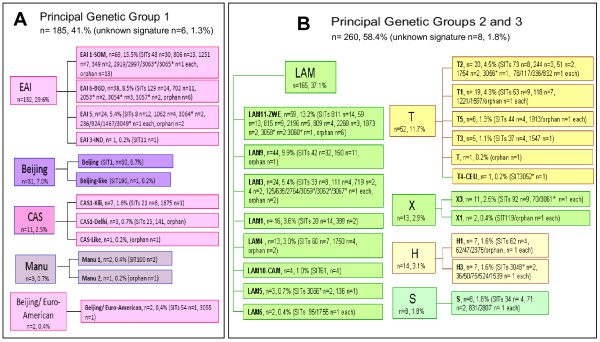
**The principal genetic groups (PGG) in Mozambique**. The figure illustrates the 4 most predominant clades in our study comprised both PGG1 and PGG2/3 lineages: LAM (PGG 2/3); ancestral EAI (PGG1); T clade (PGG 2/3); and the globally-emerging Beijing clone (PGG1).

When the spoligotyping results and clade definitions were linked to the distribution of clinical isolates within Principal Genetic Group (PGG) 1 versus PGG2/3 (characterized by the lack of spacers 33-36), it was evident that 185 or 41.6% of the isolates belonged to PGG1 (ancient lineages) as compared to 260 or 58.4% to the PGG2/3 (modern lineages) (Fig 2).

If one takes the sample of clinical isolates with newly created SITs in the database and orphans as an indication of newly documented diversity of tubercle bacilli, a total of 39/185 or 21.1% PGG1 strains were newly documented as opposed to 38/260 or 14.6% PGG2/3 strains.

As illustrated in Fig 2, the 4 most predominant lineages comprised both PGG1 and PGG2/3 lineages: Latin-American Mediterranean (LAM), n = 165 or 37.1% (PGG 2/3); ancestral East African-Indian (EAI), n = 132 or 29.7% (PGG1); an evolutionary recent but yet ill-defined T clade, n = 52 or 11.7% (PGG 2/3); and the globally-emerging Beijing clone, n = 31 or 7% (PGG1). The rest of the lineages were in the following order: Haarlem (H), n = 14 or 3.1% (PGG2/3); X clade, n = 13 or 2.9% (PGG2/3); Central Asian (CAS), n = 11 or 2.5% (PGG1). Moreover, we found 5 isolates with Manu patterns (2 isolates with Manu1 pattern and 3 isolates with Manu2 pattern) or 1.1% (PGG1), that were further investigated for Region of Difference (RD) 105 polymorphism.

A high spoligotype diversity was documented for EAI, LAM and T lineages (Fig 2). Indeed, out of the 12 sublineages reported so far worldwide for the LAM clade [5], a total of 8 sublineages were present in our 1 year recruitment. A high diversity was also evidenced for other PGG1 clades (CAS), as well as PGG2/3 clades (X clade and H).

Furthermore, no *M. africanum *or *M. bovis *were found in this study.

We also attempted to describe the worldwide distribution of predominant SITs (and lineages) encountered in this study. As shown in Table 1, we observed that many of the predominant SITs in our study belonging both to ancient PGG1 strains (SIT8/EAI5, SIT48/EAI1-SOM, SIT129/EAI6-BGD, SIT702/EAI6-BGD1, SIT806/EAI1-SOM) and evolutionary recent PGG2/3 strains (SIT33/LAM3, SIT59/LAM11-ZWE, SIT92/X3, SIT811/LAM11-ZWE, SIT815/LAM11-ZWE) were more frequently present in Eastern and Southern Africa (mostly among its immediate neighbours Zimbabwe, Zambia, South Africa, Malawi, and to a lesser extent to Tanzania, Namibia, and Somalia). Furthermore, 8 lineages or sublineages in Table 1 were made-up of their prototypes in the SITVIT2 database; these concerned SIT20 for LAM1, SIT33 for LAM3, SIT42 for LAM9, SIT48 for EAI1-SOM, SIT53 for T1, SIT59 for LAM11-ZWE, and SIT92 for X3 sublineages.

**Table 1:** Description of predominant SITs (representing 8 or more strains) in our study, and their worldwide distribution

SIT (Clade)	Number (%) in this study	% in this study as compared to SITVIT2	Distribution in Regions with 5% of a given SITs *	Distribution in countries with ≥5% of a given SITs **
1 (Beijing)	30 (6.74)	0.46	AMER-N 30.72, ASIA-SE 13.92, AFRI-S 11.76, ASIA-E 11.21, ASIA-N 8.36	USA 30.65, ZAF 11.77, RUS 8.36, JPN 8.19, VNM 5.96
8 (EAI5)	12 (2.70)	10.26	AFRI-E 26.50, EURO-N 24.79, AMER-N 24.79, ASIA-W 6.84, AFRI-S 5.13	USA 24.79, DNK 13.68, MOZ 10.26, TZA 9.40, GBR 8.55, ZMB 6.84, SAU 5.13, ZAF 5.13
20 (LAM1)	14 (3.15)	2.02	AMER-S 24.68, AMER-N 24.68, AFRI-S 12.84, EURO-S 11.40, EURO-W 8.23, CARI 6.20, AFRI-E 5.05	USA 22.94, BRA 14.29, NAM 8.95, PRT 7.07, VEN 6.06
33 (LAM3)	8 (1.80)	0.83	AFRI-S 32.60, AMER-S 23.33, AMER-N 16.77, EURO-S 14.37, EURO-W 5.73	ZAF 32.60, USA 16.56, BRA 9.48, ESP 9.27, ARG 5.94, PER 5.83
42 (LAM9)	32 (7.19)	1.26	AMER-S 30.62, AMER-N 16.71, EURO-S 13.12, EURO-W 7.21, AFRI-N 5.20	USA 15.65, BRA 10.60, COL 8.08, ITA 6.90
48 (EAI1-SOM)	30 (6.74)	7.89	EURO-N 26.32, ASIA-S 21.32, EURO-W 15.00, AFRI-E 10.00, AFRI-S 9.47, ASIA-SE 5.00	DNK 15.53, BGD 14.21, NLD 12.37, ZAF 9.47, MOZ 8.95, IND 6.05, GBR 5.26
53 (T1)	9 (2.02)	0.19	AMER-N 19.91, AMER-S 14.64, EURO-W 12.97, EURO-S 10.14, ASIA-W 8.79, AFRI-S 6.03	USA 17.54, ZAF 5.89, ITA 5.19
59 (LAM11-ZWE)	13 (2.92)	3.39	AFRI-E 67.89, AFRI-S 19.06	ZMB 27.68, ZWE 20.10, ZAF 19.06, TZA 8.36
73 (T2)	8 (1.80)	4.15	AMER-N 21.24, EURO-S 19.69, AFRI-S 13.47, EURO-W 12.44, AMER-S 10.36, AFRI-E 7.25	USA 18.65, ITA 17.62, ZAF 13.47, MOZ 5.18
92 (X3)	9 (2.02)	2.34	AFRI-S 49.09, AMER-N 24.42, AMER-S 9.61, EURO-N 5.19	ZAF 49.09, USA 21.82, BRA 5.71
129 (EAI6-BGD1)	14 (3.15)	35.90	AFRI-E 58.97, AMER-S 12.82, AMER-N 12.82, EURO-W 5.13, AFRI-N 5.13	MOZ 38.46, USA 12.82, GUF 10.26, MWI 10.26, TUN 5.13
150 (LAM9)	11 (2.47)	12.36	EURO-W 33.71, AMER-S 23.60, EURO-S 17.98, AFRI-E 13.48	BEL 24.72, MOZ 12.36, PRT 10.11, FXX 8.99, BRA 8.99, ITA 6.74, ARG 6.74, VEN 5.62
702 (EAI6-BGD1)	11 (2.47)	34.38	AFRI-E 71.88, AMER-S 15.62, CARI 6.25	MOZ 34.38, MWI 28.12, BRA 12.50, ZMB 9.38, CUB 6.25
806 (EAI1-SOM)	13 (2.92)	26.53	AFRI-S 44.90, AFRI-E 34.69, AMER-N 16.33	ZAF 44.90, MOZ 30.61, USA 16.33
811 (LAM11-ZWE)	14 (3.15)	26.92	AFRI-E 51.92, AFRI-S 38.46, AMER-N 9.62	ZAF 38.46, MOZ 28.85, ZWE 15.38, USA 9.62
815 (LAM11-ZWE)	9 (2.02)	7.83	AFRI-E 73.91, AFRI-S 21.74	ZMB 54.78, ZAF 21.74, ZWE 7.83, MOZ 7.83

* Worldwide distribution is reported for regions with ≥5% of a given SITs as compared to their total number in the SITVIT2 database. The definition of macro-geographical regions and sub-regions http://unstats.un.org/unsd/methods/m49/m49regin.htm is according to the United Nations; Regions: AFRI (Africa), AMER (Americas), ASIA (Asia), EURO (Europe), and OCE (Oceania), subdivided in: E (Eastern), M (Middle), C (Central), N (Northern), S (Southern), SE (South-Eastern), and W (Western). Furthermore, CARIB (Caribbean) belongs to Americas, while Oceania is subdivided in 4 sub-regions, AUST (Australasia), MEL (Melanesia), MIC (Micronesia), and POLY (Polynesia). Note that in our classification scheme, Russia has been attributed a new sub-region by itself (Northern Asia) instead of including it among rest of the Eastern Europe. It reflects its geographical localization as well as due to the similarity of specific TB genotypes circulating in Russia (a majority of Beijing genotypes) with those prevalent in Central, Eastern and South-Eastern Asia.

** The 3 letter country codes are according to http://en.wikipedia.org/wiki/ISO_3166-1_alpha-3; countrywide distribution is only shown for SITs with ≥5% of a given SITs as compared to their total number in the SITVIT2 database.

### Geographical distribution of spoligotypes

*M. tuberculosis *genotype distribution of the predominant lineages from the South and North regions of Mozambique is illustrated in Fig 1. A comparison of spoligotype distribution among the two regions indicates that the LAM, EAI and T lineages were common across the country, while the Beijing lineage was found to be more common in the South 27/282 (9.6%) compared to the North 4/163 (2.5%).

### RD105 analysis of Manu pattern isolates

Since the Manu2 pattern (all spacers present except spacers 33 and 34) may eventually correspond to a mixed pattern due to concomitant Beijing and Euro-American lineage strains (the latter comprising H, LAM, X, and T lineages per spoligotyping defined clades), we further investigated the five Manu pattern isolates for the presence of RD105. In one of the Manu2 pattern samples (MOZ12007E00540) we observed a 2 banded RD105 pattern, yielding an intact PCR product (characteristic of non-Beijing strains) as well as a deleted product (characteristic of Beijing strains), indicating a mixed infection. The second Manu2 pattern sample (MOZ12007E00126) showed only one band, with the RD 105 deletion, indicating that the original culture contained a mix of two strains (Beijing and non-Beijing) which on subculture and subsequent RD analysis had retained only the Beijing strain. The third Manu2 pattern sample (MOZ12007E00153) yielded a one band pattern with an intact RD105 product. We therefore conclude that two Manu2 patterns may be attributed to mixed infections by Beijing (all spacers absent except sp. 35 to 43), and T1 sublineage strain (characterized by the presence of sp. 1 to 32, and sp. 37 to 43), or due to simultaneous presence of Beijing and T2, or T2_Uganda sublineages (T2 being characterized by the presence of sp. 1 to 32, sp. 37 to 39, and sp. 41 to 43; T2_Uganda being characterized by the presence of sp. 1 to 32, sp. 37 to 39, and sp. 41 to 42). On the other hand, the third Manu2 pattern (MOZ12007E00153) represents a true Manu2 strain.

In the two samples with Manu1 pattern we did observe the presence of the genomic region RD105.

## Discussion

This study represents the first report on the genetic diversity of circulating MTC strains in Mozambique. We found that TB lineages frequently isolated in Mozambique may be nearly equally attributed both to ancestral and evolutionary modern *M. tuberculosis *lineages with a high spoligotype diversity documented for EAI, LAM and T lineages. The spoligotype diversity within these lineages suggests that they have circulated in Mozambique for some time. Spoligotype diversity was also evidenced for other PGG1 clade (CAS) as well as PGG2/3 clades (X and H). However, the "T" genotype does not represent a clade in a strict evolutionary sense since it was defined by default to include strains that may not be classified in one of the established genotypic lineages with well-established phylogeographical specificity such as the H, LAM, CAS, and EAI lineages [5].

The wide diversity may be attributed to the extensive human movement in the country mainly due to Mozambican migration to neighbouring countries and internal migration to look for better life conditions. The structure of the TB population is determined by geography, demography and human migration.

With the exception of ubiquitous spoligotypes (such as the T clade found throughout the world), the patients in Mozambique mainly harboured *M. tuberculosis *spoligotypes prevailing in Eastern and Southern Africa. Thus, in two studies conducted in Tanzania LAM (LAM11-ZWE) and EAI were found to be abundant, although the CAS (CAS1-Kili) lineage was predominant [6,7]. In another study conducted in Zimbabwe, 23 (10.7%) of 214 isolates were LAM 9 (SIT 42) [8]. In Kenya, on the other hand, 35.6% of 73 isolates were of the CAS lineage, while 11% were LAM [9].

A study conducted in Zimbabwe, Zambia and South Africa identified a predominant group of strains (designated Southern Africa 1) in Zimbabwe and Zambia with a unique spoligotype signature where spacers 21-24, 27-30 and 33-36 were deleted [10]. In our study, 44/445 (9.9%) isolates had the mentioned signature (corresponding to LAM11_ZWE), five were orphan and 39 matched a pre-existing shared type in the SITVIT2 or were newly-created either within the present study or after a match with an orphan in the database.

A remarkable feature was the presence of the ancestral Manu lineage strains (n = 3 or 0.67%). At the time of this comparison, the SITVIT2 database contained only 261 Manu lineage isolates representing less than 0.4% clinical isolates worldwide, out of which only 29 were isolated in Africa (with the exception of Egypt, where it represented 27% of all isolates [11]), however none was yet reported from Mozambique. Furthermore, with the exception of 3 Manu1 lineage strains isolated in Tanzania, all the remaining *M. tuberculosis *strains isolated from Africa belonged to the Manu2 sublineage. Hence our study constitutes the first evidence of the presence of the Manu lineage in Mozambique. With both Beijing and Euro-American strains (lacking spacers 33-36) circulating in Mozambique, some of the Manu2 patterns on the other hand appear to result from mixed infections of Beijing and Euro-American TB. Such a mixture has been described in adjacent South Africa [12].

SIT1 corresponding to the Beijing genotype was the third most frequent single spoligotype in Mozambique. The Beijing lineage has spread globally during recent years [13,14], and is seen as an indicator strain for recent import of *M. tuberculosis *into a setting. Interestingly, only one of the 31 Beijing isolates was drug resistant (data not shown); in spite of the multidrug-resistance linked to this emerging clone worldwide. A high and increasing incidence of the Beijing lineage has been described in neighbouring South Africa. In a study conducted in Cape Town the proportion of W-Beijing strains in children increased drastically from 13 to 33% from 2000 to 2003, showing that this strain has a significant selective advantage to spread within the community [15]. In the same region, an association between the Beijing lineage and HIV has recently been reported [16]. Moreover, another study carried out in Malawi demonstrates an increase over time of the proportion of TB due to Beijing genotype strains [17].

No *M. africanum *isolates were detected. *M. africanum *is highly prevalent in West African countries, with its epicentre in Guinea Bissau [18,19] but is rarely seen in East and Southern Africa [10,20]. The *M. tuberculosis *genotype T2-Uganda (previously designated *M. africanum *subtype II) was shown to be mainly responsible for the TB epidemic in Kampala, Uganda [20], although not so common in other East African countries as Kenya [9] and the Mozambican neighbour Tanzania [7]. In our study, no strains of the *M. tuberculosis *genotype T2-Uganda [20] were found.

The total absence of *M. bovis *in this one year study is noteworthy. Although bovine TB is an important disease of cattle and other domestic animals in Mozambique, no *M. bovis*, the causative agent of bovine TB, was found. One reason could be that we have studied only sputum isolates. *M. bovis *is thought to spread through unpasteurized milk, and hence would mainly cause abdominal or disseminated TB.

This study represents a first baseline study of the *M. tuberculosis *population structure in Mozambique, a useful guide for future epidemiological studies in the country and extending the picture of global TB distribution.

## Conclusions

This study demonstrated that the TB epidemic in Mozambique is caused by a wide diversity of spoligotypes with predominance of four genotype lineages: LAM, EAI, T and Beijing. The Beijing genotype was the third most frequent single spoligotype in Mozambique.

## Methods

### Ethical considerations

Institutional permission to conduct the study was obtained from the National Bioethics Committee of the Ministry of Health in Maputo, Mozambique, reference number 148/CNBS/07. The patients were included in the resistance survey after understanding the study and having signed an informed consent. They were HIV tested after completely voluntary acceptance.

### Patients and specimens

This study included a total of 445 consecutive samples of *M. tuberculosis *isolates collected during a 1 year (2007-2008) Nation Wide Drug Resistance Surveillance study performed by the National TB Control Program of Mozambique in 40 random selected districts around the country according to WHO guide-lines [21],

Clinical specimens were processed at the individual district laboratories for smear microscopy, and the sputum samples were referred to the National Reference Laboratory for culture and drug susceptibility testing (1124 positive cultures were analysed).

For the present study, 445 consecutive isolates from new pulmonary TB cases (i.e. patients with pulmonary TB who had never been treated for TB or had been treated for less than 30 days) from patients older than ≥15 years, from seven provinces of Mozambique (Maputo City, Maputo Province, Gaza, Inhambane, Nampula, Cabo Delgado and Niassa) were afterwards re-cultured and inactivated cultures were sent to the Centre of Biotechnology of Eduardo Mondlane University, in Maputo City and to the Swedish Institute for Infectious Disease Control, in Solna, for genotyping.

Basic demographic data was collected for each patient using a standard questionnaire. Patients were offered HIV-testing, and for those consenting HIV-testing was performed.

### RD 105 polymorphism

Genomic deletion of region of difference RD105 (deleted in Beijing lineage) was analysed by PCR using primer sets as previously described [22] and the PCR products were analysed by agarose gel electrophoresis.

### Spoligotyping

Standard spoligotyping [3] was performed generally as described by Kamerbeek and colleagues using a commercially available kit (Isogen Life Science B.V., Utrecht, The Netherlands). Spoligotyping results were analysed with the BioNumerics Software ver. 5.01 (Applied Maths, Kortrijk, Belgium).

### Database comparison and geographical distribution of spoligotypes

Spoligotypes in binary format were entered in the SITVIT2 database (Pasteur Institute of Guadeloupe), which is an updated version of the previously released SpolDB4 database [5]. In this database, SIT (Spoligotype International Type) designates spoligotyping shared by two or more patient isolates, as opposed to "orphan" which designates patterns reported for a single isolate. Major phylogenetic clades were assigned according to signatures provided in SpolDB4, which defined 62 genetic lineages/sub-lineages [5]. These include specific signatures for various MTC members such as *M. bovis, M. caprae, M. microti, M. canettii, M. pinnipedii*, and *M. africanum*, as well as rules defining major lineages/sub-lineages for *M. tuberculosis sensu stricto*; these include the Beijing clade, the CAS clade and 2 sublineages, the EAI clade and 9 sublineages, the H clade and 3 sublineages, the LAM clade and 12 sublineages, the ancestral "Manu" lineage and 3 sublineages, the S clade, the IS*6110*-low-banding X clade and 3 sublineages, and an ill-defined T clade with 5 sublineages (as well as further well-characterized phylogeographical specificity for 8 additional spoligotype signatures). At the time of the present study, SITVIT2 contained more than 3000 SITs with global genotyping information on about 73,000 MTC clinical isolates from 160 countries of origin.

Worldwide distribution of predominant spoligotypes found in this study (SITs representing 8 or more strains) was further investigated using the SITVIT2 database, and was recorded for regions representing ≥5% of a given SIT as compared to their total number in the SITVIT2 database. The various macro-geographical regions and sub-regions were defined according to the specifications of the United Nations [23]. More specifically, we also studied a countrywide distribution, recorded only for countries with ≥5% of a given SIT as compared to its total number in the database (3 letter country codes were according to [24]).

The overall distribution of strains were compared according to major *M. tuberculosis *genotypic families and further linked to "ancient" and "modern" lineages of tubercle bacilli as defined by PGG based on *KatG463-gyrA95 *polymorphism [25], inferred from the reported linking of specific spoligotype patterns to PGG1, 2 or 3 [26-28].

### HIV testing

HIV testing was performed according to the recommendations by the Ministry of Health, Mozambique at the Sanitary Unit of enrolment. Two rapid HIV tests were used sequentially, Unigold Recombinant HIV (Trinity Biotech, Wicklow, Ireland) and Determine HIV-1/2 (Abbot, Tokyo, Japan). Samples were tested first with Determine and reported only when negative. Positive samples were confirmed with Unigold. All tests were performed and interpreted according to the manufacturer's instructions.

## Authors' contributions

SOV participated in the design and conduct of the study, culture and isolation of mycobacteria, molecular assay, data analysis and drafting of manuscript; AM participated in the molecular assay and critical revision of manuscript, RG participated in the molecular assay and critical revision of manuscript; SG participated in the data analysis and critical revision of manuscript; AP participated in the molecular assay and critical revision of manuscript; PSG participated in the sample and data collection and critical revision of manuscript; ZC participated in the sample and data collection and critical revision of manuscript; PM participated in the sample and data collection and critical revision of manuscript; VH participated in the data analysis and drafting the manuscript; TM participated in the isolates reculture, molecular assay and critical revision of manuscript; DMC participated in protocol of the survey preparation, rechecking of strains and the sample and data collection and critical revision of manuscript; NR participated in the data analysis and drafting the manuscript; GK participated in the conception and design of the study, general supervision of the research, and critical revision of the manuscript; TK participated in the design of the study, general supervision of the research and critical revision of the manuscript. All authors read and approved the final version of the manuscript.

## Supplementary Material

Additional file 1 **Description of the orphan strains (n = 49) and corresponding spoligotyping defined lineages**. Click here for file

Additional file 2 **Description of 98 shared types from Mozambique**. A total of 79 SITs containing 368 isolates matched a preexisting shared type (SIT) in the SITVIT2 database, whereas 19 SITs (containing 28 Isolates) were newly-created either within the present study or after a match with an orphan in the database. Click here for file
