# Supplementary material for: Molecular diversity of Mycobacterium tuberculosis isolates from patients with pulmonary tuberculosis in Mozambique
Source: BMC Microbiol. 2010 Jul 21;10:195. doi: 10.1186/1471-2180-10-195 (PMC2914001; doi:10.1186/1471-2180-10-195)
Supplement: Additional file 1 — Description of the orphan strains (n = 49) and corresponding spoligotyping defined lineages. [file 1471-2180-10-195-S1.DOC]

## Additional file 1- Description of the orphan strains (n=49) and corresponding spoligotyping defined lineages.

| **ISO Number*** | **Year** | **Spoligotype Description**** | **Octal code** | **Clade***** | **Sex/Age** |
| --- | --- | --- | --- | --- | --- |
| MOZ0120080E01637 | 2008 | □□□□□□□□□□□□□□□□□□□□□□□□□□■■■■■■□□□□■■■■■■■ | 000000001760771 | Unk | M/41 |
| MOZ0120070E00007 | 2007 | □□□□□□□□□□□□□■■■■■■■■■■■■■■■■■■■□□□□■■■■■■■ | 000037777760771 | T | M |
| MOZ0120080E01313 | 2008 | ■□□□□□□□□□□□□□□□□□□□□□■■■■■■■■■■□□□□■■■□■■■ | 400000037760731 | Unk | M/28 |
| MOZ0120080E01485 | 2008 | ■□□□□□□□□□□□□■■■■■■■■■■■■■■■□□□□■□■■■■■□□□□ | 400037777413700 | EAI5 | F/25 |
| MOZ0120070E00027 | 2007 | ■□□□□□□□□□□□□■■■■■■■■■■■■■■■□□□□■□■■■■■■■□□ | 400037777413760 | EAI5 | F/21 |
| MOZ0120080E01089 | 2008 | ■□□□□□□□□□□■■■■■■■■■□□□□■■□□□□■■□□□□■■■■■■■ | 400177606060771 | LAM11-ZWE | F/32 |
| MOZ0120070E00212 | 2007 | ■□□■□■■■■■■■■■□■■■■■□□■■■■■■□□□□■□■■■■■□■■■ | 457767637413731 | EAI1-SOM | M/56 |
| MOZ0120080E01180 | 2008 | ■□■□□□□□□■■■■■■■□■■■■■□□■■■■□□□□■□■■■■■■■■■ | 500775747413771 | EAI6-BGD1 | F/28 |
| MOZ0120080E01269 | 2008 | ■□■■■■■■■■■■■■■■■■■■□□□□■■□□□□■■□□□□■■■□■■■ | 577777606060731 | LAM11-ZWE | F/17 |
| MOZ0120070E00195 | 2007 | ■□■■■■■■■■■■■■■■■■■■■■■■□■□□□□□□□□□□□□□□□□□ | 577777772000000 | Unk | M/27 |
| MOZ0120080E00325 | 2008 | ■■□□□□□□□□□□□□□□□□■■□□□□■■□□□□■■□□□□■■□□■■■ | 600000606060631 | LAM11-ZWE | F/41 |
| MOZ0120070E00105 | 2007 | ■■□□□□□□□□□□□□□□■■■■■■■■■□□□□□□■□□□□■■■■■■■ | 600003774020771 | H1 | F/36 |
| MOZ0120070E00206 | 2007 | ■■□□□□□□□□■■■■□■■■■■■■□□■■■■□□□□■□■■■■■■■■■ | 600367747413771 | EAI6-BGD1 | M/25 |
| MOZ0120070E00232 | 2007 | ■■□□■■■■□□□■■■□■■■■■□□□□■■■■■■■■□□□□■■■■■■■ | 636167607760771 | LAM3 | M/29 |
| MOZ0120080E01421 | 2008 | ■■□■■■■□■■■■■■■■■■■■□□□□■■■■■■■□□□□□□■■□■■■ | 675777607740331 | LAM4 | F/49 |
| MOZ0120070E00111 | 2007 | ■■□■■■■■■■■□■■■■■■■□□□□■■■■■■■■■□□□□■■■■■■■ | 677677417760771 | Unk | F/22 |
| MOZ0120070E00092 | 2007 | ■■■□□□□□□□□□■■□■■□■■■■■■■■■■□□□□□□□□■■■■■■■ | 700066777400771 | Unk | F/22 |
| MOZ0120080E00322 | 2008 | ■■■□□□□□□■■■■■■■■■■■■■□□■■■■□□□□■□■■■■■□■■■ | 700777747413731 | EAI1-SOM | M/67 |
| MOZ0120070E00149 | 2007 | ■■■□□□□□■■■■■■□■■■■■□□□□■■■■□□□□■□■■■■■□■■■ | 701767607413731 | EAI1-SOM | M/40 |
| MOZ0120070E00173 | 2007 | ■■■□□□□■□■■■■■□■■■■■■■□□□□□□□□□□□□■■■■■■■■■ | 702767740003771 | CAS1-Delhi | M/63 |
| MOZ0120070E00028 | 2007 | ■■■□□□□■■■■□□□□□□□□□□□□□□□□□□□□□□□■■■■■■■■■ | 703600000003771 | CAS_LIKE | M/26 |
| MOZ0120070E00032 | 2007 | ■■■□□■■■■■■□□□□□■■■■■■□■□■□□□□□□□□□□□□□□□□□ | 717603752000000 | Unk | M/38 |
| MOZ0120070E00112 | 2007 | ■■■□□■■■■■■□□□□□■■■■■■□■■■■■□□□□■□□□□■■■■■■ | 717603757410371 | EAI6-BGD1 | F/25 |
| MOZ0120070E00181 | 2007 | ■■■■□■■□■■□■■■□■■■■■□■■■■■■■□□□□□□■□■■□□■■■ | 755567677402631 | Unk | M/65 |
| MOZ0120070E00201 | 2007 | ■■■■□■■■■■□□□■□■■■■■□□□□■■■■□□□□■□■■■■■□■■■ | 757427607413731 | EAI1-SOM | M/43 |
| MOZ0120080E00321 | 2008 | ■■■■□■■■■■■■■■□■■■■■□■■■■■■■□□□□■□■■■■■□■■■ | 757767677413731 | EAI1-SOM | M/39 |
| MOZ0120070E00235 | 2007 | ■■■■□■■■■■■■■■□■■■■■■■■■■■■■□□□□■□■■■■■□■■■ | 757767777413731 | EAI1-SOM | M/23 |
| MOZ0120080E00580 | 2008 | ■■■■□■■■■■■■■■■■□■■■■■■■■■■■□□□□■□■■■■■□■■■ | 757775777413731 | EAI1-SOM | F/18 |
| MOZ0120080E01486 | 2008 | ■■■■□■■■■■■■■■■■■■■□■■■■■■■■□□□□■□■■■■■□■■■ | 757777577413731 | EAI1-SOM | F/50 |
| MOZ0120080E01465 | 2008 | ■■■■■□■■■■■■■■■■■■■■■■□□□■■■□□□□■□■■■■■■■■■ | 767777743413771 | EAI6-BGD1 | F/18 |
| MOZ0120070E00199 | 2007 | ■■■■■■□□■■□■■■□■■■■■■■□■■■■■■■■■□□□□■■□■■■■ | 771567757760671 | T5 | M/25 |
| MOZ0120070E00171 | 2007 | ■■■■■■■□□□□□□■□■■■■■■■■■■■■■□□□□■□■■■■■□■■■ | 774027777413731 | EAI1-SOM | F/50 |
| MOZ0120070E00159 | 2007 | ■■■■■■■□■■□■■■□■■■■■□□□□■■■■■■■■□□□□■■■■■■■ | 775567607760771 | LAM9 | M/35 |
| MOZ0120070E00183 | 2007 | ■■■■■■■□■■□■■■□■■■■■□■■■■■■■■■■■□□□□■■■■■■■ | 775567677760771 | T1 | F |
| MOZ0120070E00234 | 2007 | ■■■■■■■■□□□■■■□■■■■■□□□□■■■■■■■■□□□□■□□□□□■ | 776167607760401 | LAM3 | M/30 |
| MOZ0120080E01371 | 2008 | ■■■■■■■■■□□□□□□□□□□□□□□□□□□□□□■■□□□□■■■■■■■ | 777000000060771 | Unk | F/25 |
| MOZ0120070E00017 | 2007 | ■■■■■■■■■■□■■■□■■□■■■■■■■■■■■■■■□□□□■■■■■■■ | 777566777760771 | X1 | M/28 |
| MOZ0120080E01456 | 2008 | ■■■■■■■■■■□■■■■■■■■■□□□□■■□□□□■■□□□□■■■□■■■ | 777577606060731 | LAM11-ZWE | F/35 |
| MOZ0120070E00087 | 2007 | ■■■■■■■■■■■□□□□□□■■■■■□■■■■■□□□□■□■■□■■■■■■ | 777601757413371 | EAI6-BGD1 | F/21 |
| MOZ0120080E01156 | 2008 | ■■■■■■■■■■■□□■■■□■■■■■□■■■■■□□□□■□■■□■■■■■■ | 777635757413371 | EAI6-BGD1 | M/45 |
| MOZ0120070E00157 | 2007 | ■■■■■■■■■■■■□■□■■■■■■■■■■■■■□□□□■□■■■■■□■■■ | 777727777413731 | EAI1-SOM | M/65 |
| MOZ0120070E00119 | 2007 | ■■■■■■■■■■■■■■□■□■■■□□□□■■□□□□■■□□□□■■□□■■■ | 777765606060631 | LAM11-ZWE | M/37 |
| MOZ0120070E00029 | 2007 | ■■■■■■■■■■■■■■□■■■■■□□□□■■□□□□■■□□□□■■□□■■■ | 777767606060631 | LAM11-ZWE | F/60 |
| MOZ0120070E00208 | 2007 | ■■■■■■■■■■■■■■□■■■■■■□□■■■■■□□□□■□■■■■■□■■■ | 777767717413731 | EAI1-SOM | M/39 |
| MOZ0120070E00182 | 2007 | ■■■■■■■■■■■■■■□■■■■■■■■■■■■■□□□□□□□□■■■■■■■ | 777767777400771 | Unk | M/60 |
| MOZ0120080E00459 | 2008 | ■■■■■■■■■■■■■■■■□■■■□□□□■■□□□□□□□□□□■■■□■■■ | 777775606000731 | LAM4 | M/57 |
| MOZ0120080E00583 | 2008 | ■■■■■■■■■■■■■■■■■■■■■□□■■■■■□□□□■□■■■■■□■■■ | 777777717413731 | EAI1-SOM | F/41 |
| MOZ0120070E00106 | 2007 | ■■■■■■■■■■■■■■■■■■■■■■□■■■■■□□□□■□■■■■■□■■■ | 777777757413731 | EAI1-SOM | F/16 |
| MOZ0120070E00153 | 2007 | ■■■■■■■■■■■■■■■■■■■■■■■■■■■■□□■■□□■■■■■□■■■ | 777777777463731 | Manu2 | F/30 |

* The 16 digit ISO number in the SITVIT2 database; the last 6 digits beginning with letter E represent the laboratory number of the isolate.

** The black and white boxes indicate the presence and absence, respectively, of the specific spacer at positions 1–43 in the DR locus.

*** Clade designations according to SITVIT2 using revised SpolDB4 rules; Unk: Unknown patterns within any of the major clades described in SITVIT2.
