# Supplementary material for: Molecular diversity of Mycobacterium tuberculosis isolates from patients with pulmonary tuberculosis in Mozambique
Source: BMC Microbiol. 2010 Jul 21;10:195. doi: 10.1186/1471-2180-10-195 (PMC2914001; doi:10.1186/1471-2180-10-195)
Supplement: Additional file 2 — Description of 98 shared types from Mozambique. A total of 79 SITs containing 368 isolates matched a preexisting shared type (SIT) in the SITVIT2 database, whereas 19 SITs (containing 28 Isolates) were newly-created either within the present study or after a match with an orphan in the database. [file 1471-2180-10-195-S2.DOC]

**Additional file 2. Description of 98 shared types from Mozambique**

| **SIT*** | **Spoligotype Description**** | **Octal Number** | **Number (%) in this study** | **% in this study as compared to database** | **Clade***** | **Clustered vs. unique patterns****** |
| --- | --- | --- | --- | --- | --- | --- |
| 1 | □□□□□□□□□□□□□□□□□□□□□□□□□□□□□□□□□□■■■■■■■■■ | 000000000003771 | 30 (6.74) | 0.46 | Beijing | Clustered |
| 4 | □□□□□□□□□□□□□□□□□□□□□□□□■■■■■■■■□□□□■■■■■■■ | 000000007760771 | 2 (0.45) | 0.74 | LAM3 | Clustered |
| 8 | ■□□□□□□□□□□□□■■■■■■■■■■■■■■■□□□□■□■■■■■■■■■ | 400037777413771 | 12 (2.70) | 10.26 | EAI5 | Clustered |
| 11 | ■□□■■■■■■■■■■■■■■■■■■■■■■■■■□□□□■□■■□□□■■■■ | 477777777413071 | 1 (0.22) | 0.21 | EAI3-IND | Unique |
| 20 | ■■□■■■■■■■■■■■■■■■■■□□□□■■■■■■■■□□□□■■■■■■■ | 677777607760771 | 14 (3.15) | 2.02 | LAM1 | Clustered |
| 21 | ■■■□□□□■■□■■■■■■■■■□□□□□□□□□□□□□□□□■■■■■■■■ | 703377400001771 | 6 (1.35) | 1.92 | CAS1-Kili | Clustered |
| 25 | ■■■□□□□■■■■■■■■■■■■■■■□□□□□□□□□□□□■■□□■■■■■ | 703777740003171 | 1 (0.22) | 0.30 | CAS1-Delhi | Unique |
| 33 | ■■■■■■■■□□□■■■■■■■■■□□□□■■■■■■■■□□□□■■■■■■■ | 776177607760771 | 8 (1.80) | 0.83 | LAM3 | Clustered |
| 34 | ■■■■■■■■□□■■■■■■■■■■■■■■■■■■■■■■□□□□■■■■■■■ | 776377777760771 | 4 (0.90) | 0.56 | S | Clustered |
| 36 | ■■■■■■■■■■■■□■■■■■■■■■■■■■■■■■□■□□□□■■■■■■■ | 777737777720771 | 1 (0.22) | 1.04 | H3 | Unique |
| 37 | ■■■■■■■■■■■■□■■■■■■■■■■■■■■■■■■■□□□□■■■■■■■ | 777737777760771 | 4 (0.90) | 1.35 | T3 | Clustered |
| 42 | ■■■■■■■■■■■■■■■■■■■■□□□□■■■■■■■■□□□□■■■■■■■ | 777777607760771 | 32 (7.19) | 1.26 | LAM9 | Clustered |
| 44 | ■■■■■■■■■■■■■■■■■■■■■■□■■■■■■■■■□□□□■■■■■■■ | 777777757760771 | 4 (0.90) | 2.23 | T5 | Clustered |
| 47 | ■■■■■■■■■■■■■■■■■■■■■■■■■□□□□□□■□□□□■■■■■■■ | 777777774020771 | 1 (0.22) | 0.08 | H1 | Unique |
| 48 | ■■■■■■■■■■■■■■■■■■■■■■■■■■■■□□□□■□■■■■■□■■■ | 777777777413731 | 30 (6.74) | 7.89 | EAI1-SOM | Clustered |
| 50 | ■■■■■■■■■■■■■■■■■■■■■■■■■■■■■■□■□□□□■■■■■■■ | 777777777720771 | 1 (0.22) | 0.04 | H3 | Unique |
| 51 | ■■■■■■■■■■■■■■■■■■■■■■■■■■■■■■■■□□□□■■■□□□□ | 777777777760700 | 2 (0.45) | 0.91 | T2 | Clustered |
| 53 | ■■■■■■■■■■■■■■■■■■■■■■■■■■■■■■■■□□□□■■■■■■■ | 777777777760771 | 9 (2.02) | 0.19 | T1 | Clustered |
| 54 | ■■■■■■■■■■■■■■■■■■■■■■■■■■■■■■■■□□■■■■■■■■■ | 777777777763771 | 1 (0.22) | 0.60 | Beijing and Euro-American | Unique |
| 59 | ■■■■■■■■■■■■■■■■■■■■□□□□■■□□□□■■□□□□■■■■■■■ | 777777606060771 | 13 (2.92) | 3.39 | LAM11-ZWE | Clustered |
| 60 | ■■■■■■■■■■■■■■■■■■■■□□□□■■■■■■■■□□□□■■■□■■■ | 777777607760731 | 7 (1.57) | 2.36 | LAM4 | Clustered |
| 61 | ■■■■■■■■■■■■■■■■■■■■■■□□□■■■■■■■□□□□■■■■■■■ | 777777743760771 | 4 (0.90) | 0.62 | LAM10-CAM | Clustered |
| 62 | ■■■■■■■■■■■■■■■■■■■■■■■■■□□□□□□■□□□□■■■□■■■ | 777777774020731 | 4 (0.90) | 0.97 | H1 | Clustered |
| 70 | ■■■□□□□□□□□□■■■■■□■■■■■■■■■■■■■■□□□□■■□■■■■ | 700076777760671 | 1 (0.22) | 0.88 | X3 | Unique |
| 71 | ■■■■■■■■□□■■□■■■■■■■■■■■■■■■■■■■□□□□■■■■■■■ | 776337777760771 | 2 (0.45) | 2.90 | S | Clustered |
| 73 | ■■■■■■■■■■■■□■■■■■■■■■■■■■■■■■■■□□□□■■■□■■■ | 777737777760731 | 8 (1.80) | 4.15 | T2 | Clustered |
| 75 | ■■■■■■■■■■■■■■□■■■■■■■■■■■■■■■□■□□□□■■■■■■■ | 777767777720771 | 1 (0.22) | 2.08 | H3 | Unique |
| 78 | ■■■■■■■■■■■■■■■■■■■■■■■■■■■■■■■■□□□□■■■□□■■ | 777777777760711 | 1 (0.22) | 1.89 | T2 | Unique |
| 92 | ■■■□□□□□□□□□■■■■■□■■■■■■■■■■■■■■□□□□■■■■■■■ | 700076777760771 | 9 (2.02) | 2.34 | X3 | Clustered |
| 95 | ■■■■■■■■■■■■■■■■■■■■□□□□■■■■□■■■□□□□■■■□■■■ | 777777607560731 | 1 (0.22) | 2.63 | LAM6 | Unique |
| 100 | ■■■■■■■■■■■■■■■■■■■■■■■■■■■■■■■■■□■■■■■■■■■ | 777777777773771 | 2 (0.45) | 2.99 | MANU1 | Clustered |
| 111 | ■■■■■■■■□□□■■■□■■■■■□□□□■■■■■■■■□□□□■■■■■■■ | 776167607760771 | 4 (0.90) | 14.81 | LAM3 | Clustered |
| 117 | ■■■■■■■■■■■■■■□■■■■■■■■■■■■■■■■■□□□□■■■□■■■ | 777767777760731 | 1 (0.22) | 4.55 | T2 | Unique |
| 118 | ■■■■■■■■■■■■■■□■■■■■■■■■■■■■■■■■□□□□■■■■■■■ | 777767777760771 | 7 (1.57) | 5.98 | T1 | Clustered |
| 119 | ■■■■■■■■■■■■■■■■■□■■■■■■■■■■■■■■□□□□■■■■■■■ | 777776777760771 | 1 (0.22) | 0.10 | X1 | Unique |
| 125 | □□□□□□□□□□□□□□□□□□□□□□□□■■■■■■■■□□□□■■■□■■■ | 000000007760731 | 1 (0.22) | 1.52 | LAM3 | Unique |
| 129 | ■■■□□□□□□■■■■■■■■■■■■■□□■■■■□□□□■□■■■■■■■■■ | 700777747413771 | 14 (3.15) | 35.90 | EAI6-BGD1 | Clustered |
| 136 | ■■■■■■■■■■■□□□□□■■■□□□□□■□■■■■■■□□□□■□□■■■■ | 777603405760471 | 1 (0.22) | 2.44 | LAM5 | Unique |
| 141 | ■■■□□□□■■■■■■■□■■■■■■■□□□□□□□□□□□□■■■■■■■■■ | 703767740003771 | 1 (0.22) | 5.88 | CAS1-Delhi | Unique |
| 150 | ■■■■■■■■■■■■■■□■■■■■□□□□■■■■■■■■□□□□■■■■■■■ | 777767607760771 | 11 (2.47) | 12.36 | LAM9 | Clustered |
| 190 | □□□□□□□□□□□□□□□□□□□□□□□□□□□□□□□□□□■■■■■□■■■ | 000000000003731 | 1 (0.22) | 1.14 | Beijing | Unique |
| 236 | ■■■■■■■■■■■■■■■■■■■■■■■■■■■■□□□□■□■■■■■■■■■ | 777777777413771 | 1 (0.22) | 0.72 | EAI5 | Unique |
| 244 | ■■■■■■■■■■■■■■■■■■■■■■■■■■■■■■■■□□□□■■□□□□■ | 777777777760601 | 3 (0.67) | 3.23 | T2 | Clustered |
| 336 | ■■■■■■■■■■■■■■■■■□■■■■■■■■■■■■■■□□□□■■■□■■■ | 777776777760731 | 1 (0.22) | 1.47 | T2 | Unique |
| 349 | ■■■■■■■■■■■■□■■■■■■■■■■■■■■■□□□□■□■■■■■□■■■ | 777737777413731 | 2 (0.45) | 8.33 | EAI1-SOM | Clustered |
| 389 | ■■□■■■■■■■■■■■□■■■■■□□□□■■■■■■■■□□□□■■■■■■■ | 677767607760771 | 2 (0.45) | 11.76 | LAM1 | Clustered |
| 524 | ■■■■■■■■■■■■■■■■■■■■■■■■■■■■■■□■□□□□■■■□□■■ | 777777777720711 | 1 (0.22) | 9.09 | H3 | Unique |
| 563 | ■■■■■■■■■■■■□■■■■■■■■■■■□□□□□□□□□□□□□□□■■■■ | 777737770000071 | 2 (0.45) | 16.67 | Unk | Clustered |
| 635 | □□□□□□□□□□□□□□□□□□□□□□□□■■■■□■■■□□□□■■■■■■■ | 000000007560771 | 1 (0.22) | 12.50 | LAM3 | Unique |
| 702 | ■■■□□□□□□■■■■■■■□■■■■■□□■■■■□□□□■□■■■■■■■■■ | 700775747413771 | 11 (2.47) | 34.38 | EAI6-BGD1 | Clustered |
| 719 | ■■■■■■■■□□□■■■■■■■■□□□□□■■■■■■■■□□□□■■■■■■■ | 776177407760771 | 2 (0.45) | 2.30 | LAM3 | Clustered |
| 780 | ■■■■■■■■■■■■■■■■■■■■■■■■■■■■■□□□□□□□■■■■■■■ | 777777777600771 | 1 (0.22) | 11.11 | Unk | Unique |
| 806 | ■■■■□■■■■■■■■■■■■■■■■■■■■■■■□□□□■□■■■■■□■■■ | 757777777413731 | 13 (2.92) | 26.53 | EAI1-SOM | Clustered |
| 809 | ■■■■■■■■■■■■■■■■□■■■□□□□■■□□□□■■□□□□■■■■■■■ | 777775606060771 | 4 (0.90) | 50.00 | LAM11-ZWE | Clustered |
| 811 | ■■■■■■■■■■■■■■■■■■■■□□□□■□□□□□■■□□□□■■■□■■■ | 777777604060731 | 14 (3.15) | 26.92 | LAM11-ZWE | Clustered |
| 815 | ■■■■■■■■■■■■■■■■■■■■□□□□■■□□□□■■□□□□■■■□■■■ | 777777606060731 | 9 (2.02) | 7.83 | LAM11-ZWE | Clustered |
| 831 | ■■■■■■■■□□■■■■□■■■■■■■■■■■■■■■■■□□□□■■■■■■■ | 776367777760771 | 1 (0.22) | 10.00 | S | Unique |
| 832 | ■■■■■■■■■■■■□■□■■■■■■■■■■■■■■■■■□□□□■■■□■■■ | 777727777760731 | 1 (0.22) | 14.29 | T2 | Unique |
| 924 | ■■■■■■■■■■■□□□□□□□□□□□□□■■■■□□□□■□■■□■■■■■■ | 777600007413371 | 1 (0.22) | 5.56 | EAI5 | Unique |
| 1062 | ■■■□□□□□□■■■■■■■□□□□□□□□■■■■□□□□■□■■■■■■■■■ | 700774007413771 | 4 (0.90) | 50.00 | EAI5 | Clustered |
| 1221 | ■■■■□■■■■■■■■■□■■■■■■■■■■■■■■■■■□□□□■■■■■■■ | 757767777760771 | 1 (0.22) | 33.33 | T1 | Unique |
| 1251 | ■■■■■■■■■■■■■■□■■■■■■■■■■■■■□□□□■□■■■■■□■■■ | 777767777413731 | 7 (1.57) | 77.78 | EAI1-SOM | Clustered |
| 1467 | ■□□■■■■■■■■■■■■■■■■■■■■■■■■■□□□□■□□□■■■■■■■ | 477777777410771 | 1 (0.22) | 20.00 | EAI5 | Unique |
| 1539 | ■■■■■■□■■■■■■■■■■■■■■■■■■■■■■■□■□□□□■■■■■■■ | 773777777720771 | 1 (0.22) | 7.14 | H3 | Unique |
| 1547 | ■■■■■■■■■■■■□■□■■■■■■■■■■■■■■■■■□□□□■■■■■■■ | 777727777760771 | 1 (0.22) | 16.67 | T3 | Unique |
| 1597 | ■■■□□□■■■■■■■■■■■■■■■■■■■■■■■■■■□□□□■■■■■■■ | 707777777760771 | 1 (0.22) | 11.11 | T1 | Unique |
| 1675 | ■■■□□□□■■□■■■■□■■■■□□□□□□□□□□□□□□□□■■■■■■■■ | 703367400001771 | 1 (0.22) | 6.25 | CAS1-Kili | Unique |
| 1750 | ■■■■■■■■■■■■■■□■■■■■□□□□■■■■■■■■□□□□■■■□■■■ | 777767607760731 | 4 (0.90) | 50.00 | LAM4 | Clustered |
| 1754 | ■■■■■■■■■■■■■■□■■■■■■■■■■■■■■■■■□□□□■■□□□□■ | 777767777760601 | 2 (0.45) | 28.57 | T2 | Clustered |
| 1755 | ■■□■■■■■■■■■■■■■■■■■□□□□■■■■□■■■□□□□■■■■■■■ | 677777607560771 | 1 (0.22) | 12.50 | LAM6 | Unique |
| 1873 | ■■■■■■■■■■■■■■■■□□■■□□□□■■□□□□■■□□□□■■■□■■■ | 777774606060731 | 2 (0.45) | 9.09 | LAM11-ZWE | Clustered |
| 1913 | ■■■■■■■■■■■■■■□■■■■■■■□■■■■■■■■■□□□□■■■■■■■ | 777767757760771 | 1 (0.22) | 33.33 | T5 | Unique |
| 2196 | ■■■■■■■■■■■■■■■■□■■■□□□□■■□□□□■■□□□□■■■□■■■ | 777775606060731 | 5 (1.12) | 38.46 | LAM11-ZWE | Clustered |
| 2268 | ■■■■■■■■■■■■■■□■■■■■□□□□■■□□□□■■□□□□■■■■■■■ | 777767606060771 | 3 (0.67) | 42.86 | LAM11-ZWE | Clustered |
| 2375 | ■■■■■□■■■■■■■■■■■■■■■■■■■□□□□□□■□□□□■■■■■■■ | 767777774020771 | 1 (0.22) | 9.09 | H1 | Unique |
| 2764 | ■■■■■■■■□□□■■■□■■■■■□□□□■■■■■■■■□□□□■■■□■■■ | 776167607760731 | 1 (0.22) | 33.33 | LAM3 | Unique |
| 2807 | ■■■■■■■■□□■■■■□■■■■■■■■■■■■■■■■■□□□□■■■□■■■ | 776367777760731 | 1 (0.22) | 25.00 | S | Unique |
| 2919 | ■□□□□□□□□□□□□■■■■■■■■■■■■■■■□□□□■□■■■■■□■■■ | 400037777413731 | 1 (0.22) | 33.33 | EAI1-SOM | Unique |
| 2997 | ■■■■□■□■■■■■■■■■■■■■■■■■■■■■□□□□■□■■■■■□■■■ | 753777777413731 | 1 (0.22) | 12.50 | EAI1-SOM | Unique |
| 3048* | ■■■■■■■■■■■■■■■■■■■■□□□□■■□□□□□■□□□□■■■□■■■ | 777777606020731 | 2 (0.45) | 100.00 | H3 | Clustered |
| 3049* | ■■■■■■■■■■■■■■□■■■■■■■■■■■■■□□□□■□■■■■□□■■■ | 777767777413631 | 1 (0.22) | 50.00 | EAI5 | Unique |
| 3050* | ■■■□■□□□□□□□□□□□□■■□□□□□□□□□□□□□□□□□□□■■■■■ | 720001400000171 | 2 (0.45) | 100.00 | Unk | Clustered |
| 3052* | ■■■■■■■■■■■■■■□■■■□■■■□□■■■■■■■■□□□□■□□■■■■ | 777767347760471 | 1 (0.22) | 50.00 | T4-CEU1 | Unique |
| 3053* | ■■■□□□□□□■■■■■□■□■■■■■□□■■■■□□□□■□■■■■■■■■■ | 700765747413771 | 2 (0.45) | 100.00 | EAI6-BGD1 | Clustered |
| 3054* | ■■■■■■■■■■■□■■■■□■■■■■□■■■■■□□□□■□■■□■■■■■■ | 777675757413371 | 3 (0.67) | 75.00 | EAI6-BGD1 | Clustered |
| 3055* | ■■■□□□□□□□□□■■■■■□■■■■■■■■■■■■■■□□■■■■■■■■■ | 700076777763771 | 1 (0.22) | 50.00 | Beijing and Euro-American | Unique |
| 3056* | ■■■■■■■■■■■■■■■■■■■■■■■■■■■■□■■■□□□□■■■□■■■ | 777777777560731 | 1 (0.22) | 50.00 | T2 | Unique |
| 3057* | ■■■□□□□□□■■■■■□■■■■■■■□□■■■■□□□□■□■■■■■■■■■ | 700767747413771 | 2 (0.45) | 100.00 | EAI6-BGD1 | Clustered |
| 3058* | ■■■■■■■■■■■■■■□■□■■■□□□□■■□□□□■■□□□□■■■□■■■ | 777765606060731 | 2 (0.45) | 100.00 | LAM11-ZWE | Clustered |
| 3059* | ■■■■■■■□□□□■■■□■■■■■□□□□■■■■■■■■□□□□■■■■■■■ | 774167607760771 | 1 (0.22) | 50.00 | LAM3 | Unique |
| 3060* | ■■□□□□□□□□□□□□□□□□■■□□□□■■□□□□■■□□□□■■■□■■■ | 600000606060731 | 1 (0.22) | 50.00 | LAM11-ZWE | Unique |
| 3061* | ■■■□□□□□□□□□■■■■■□■■■■■■■■■■■■■■□□□□■■□■■□■ | 700076777760661 | 1 (0.22) | 50.00 | X3 | Unique |
| 3062* | ■■■■■■■■□□□■■■□■■■■□□□□□■■■■■■■■□□□□■■■■■■■ | 776167407760771 | 1 (0.22) | 50.00 | LAM3 | Unique |
| 3063* | ■■■■□■■■■■■■■■■■■■■■□□□□■■■■□□□□■□■■■■■□■■■ | 757777607413731 | 1 (0.22) | 50.00 | EAI1-SOM | Unique |
| 3064* | ■■■■■■■■■■■■■■■■■■□■■■■■■■■■□□□□■□□□□□□■■■■ | 777777377410071 | 2 (0.45) | 100.00 | EAI5 | Clustered |
| 3065* | ■■■■□■■■■■■■■■■■■■■■■□■■■■■■□□□□■□■■■■■□■■■ | 757777737413731 | 1 (0.22) | 50.00 | EAI1-SOM | Unique |
| 3066* | ■■■■■■■■■■■■□■■■■□■■□□□□■■■■■■■■□□□□■■■■■■■ | 777736607760771 | 2 (0.45) | 100.00 | LAM5 | Clustered |
| 3067* | □□□□□□□□□□□□□□□□□□□□□□□□■■■■■■■■□□□□■■■■□■■ | 000000007760751 | 1 (0.22) | 50.00 | LAM3 | Unique |

* SIT followed by an asterisk indicates "newly created shared-type" (n=19) due to 2 or more strains belonging to an identical new pattern within this study (SIT designations followed by number of strains : SIT3048, n=2; SIT3050, n=2; n=5; SIT3053, n=2; SIT3057, n=2; SIT3058, n=2; SIT3064, n=2; SIT3066, n=2), or a unique strain from this study matching with another orphan in the database (SIT3049 matched an orphan from Thailand; SIT3052 matched an orphan from Italy; SIT3055 matched an orphan from USA; SIT3056, SIT3060, and SIT3062 matched an orphan each from South Africa; SIT3059 matched an orphan from Brazil; SIT3061 and SIT3065 matched an orphan each from Great Britain; SIT3063 matched an orphan from Zambia; SIT3067 matched an orphan from Argentina; and lastly, SIT3054 was created by 3 strains belonging to an identical new pattern within this study that also matched an orphan from Brazil.

^**^ The black and white boxes indicate the presence and absence, respectively, of the specific spacer at positions 1–43 in the DR locus.

** Clade designations according to SITVIT2 using revised SpolDB4 rules; Unk: Unknown patterns within any of the major clades described in SITVIT2

*** Clustered strains correspond to a similar spoligotype pattern shared by 2 or more strains “within this study”; as opposed to unique strains harboring a spoligotype pattern that does not match with another strain from this study. Unique strains matching a preexisting pattern in the SITVIT2 database are classified as SITs, whereas in case of no match, they are designated as “orphan” (see Table 1).
